# Correlation of admissions statistics to graduate student success in medical physics

**DOI:** 10.1120/jacmp.v15i1.4451

**Published:** 2014-01-06

**Authors:** Jay Burmeister, Erin McSpadden, Joseph Rakowski, Adrian Nalichowski, Mark Yudelev, Michael Snyder

**Affiliations:** ^1^ Department of Radiation Oncology Wayne State University School of Medicine Detroit MI; ^2^ Karmanos Cancer Center Gershenson R.O.C. Detroit MI; ^3^ McLaren Cancer Institute – Macomb M. Clemens MI USA

**Keywords:** education

## Abstract

The purpose of this work is to develop metrics for evaluation of medical physics graduate student performance, assess relationships between success and other quantifiable factors, and determine whether graduate student performance can be accurately predicted by admissions statistics. A cohort of 108 medical physics graduate students from a single institution were rated for performance after matriculation based on final scores in specific courses, first year graduate Grade Point Average (GPA), performance on the program exit exam, performance in oral review sessions, and faculty rating. Admissions statistics including matriculating program (MS vs. PhD); undergraduate degree type, GPA, and country; graduate degree; general and subject GRE scores; traditional vs. nontraditional status; and ranking by admissions committee were evaluated for potential correlation with the performance metrics. GRE verbal and quantitative scores were correlated with higher scores in the most difficult courses in the program and with the program exit exam; however, the GRE section most correlated with overall faculty rating was the analytical writing section. Students with undergraduate degrees in engineering had a higher faculty rating than those from other disciplines and faculty rating was strongly correlated with undergraduate country. Undergraduate GPA was not statistically correlated with any success metrics investigated in this study. However, the high degree of selection on GPA and quantitative GRE scores during the admissions process results in relatively narrow ranges for these quantities. As such, these results do not necessarily imply that one should not strongly consider traditional metrics, such as undergraduate GPA and quantitative GRE score, during the admissions process. They suggest that once applicants have been initially filtered by these metrics, additional selection should be performed via the other metrics shown here to be correlated with success. The parameters used to make admissions decisions for our program are accurate in predicting student success, as illustrated by the very strong statistical correlation between admissions rank and course average, first year graduate GPA, and faculty rating (p<0.002). Overall, this study indicates that an undergraduate degree in physics should not be considered a fundamental requirement for entry into our program and that within the relatively narrow range of undergraduate GPA and quantitative GRE scores of those admitted into our program, additional variations in these metrics are not important predictors of success. While the high degree of selection on particular statistics involved in the admissions process, along with the relatively small sample size, makes it difficult to draw concrete conclusions about the meaning of correlations here, these results suggest that success in medical physics is based on more than quantitative capabilities. Specifically, they indicate that analytical and communication skills play a major role in student success in our program, as well as predicted future success by program faculty members. Finally, this study confirms that our current admissions process is effective in identifying candidates who will be successful in our program and are expected to be successful after graduation, and provides additional insight useful in improving our admissions selection process.

PACS number: 01.40.‐d

## I. INTRODUCTION

Graduate programs in medical physics annually review numerous applicants for training positions within MS and PhD programs. Effective identification of applicants who will be successful students and future medical physicists is important not only for the educational programs themselves, but also for our profession and our patients. The number of graduate training positions available within the field of medical physics is relatively small in comparison to most other disciplines, as is the number of professional positions within the field. It is important for our profession to attempt to identify the highest quality candidates for this small number of graduate training spots. It has recently been argued that the number of graduate training spots should be reduced.^(1)^ Such a reduction would make it even more difficult to assure that the most successful candidates matriculate into medical physics graduate programs.

Selecting the most suitable candidates for graduate education requires significant resources from academic departments. Faculty time required to evaluate applications and make admissions decisions is substantial, and there is considerable cost to the department and/or candidate for on‐site interviews. Evaluating the performance of graduate students and attempting to correlate this performance with admissions statistics would be of significant benefit both in maximizing the quality of medical physics graduates and minimizing the resources expended in doing so. We have attempted to apply established metrics of student success to a cohort of over 100 medical physics graduate students from a single institution spanning nearly a decade, and to determine whether statistically significant predictors of success can be determined from their admissions statistics.

## II. MATERIALS AND METHODS

This study evaluates the admissions statistics and graduate school performance of 108 graduate students matriculating into the MS and PhD programs in Medical Physics within the Wayne State University School of Medicine between 2004 and 2011. Among the admissions statistics evaluated were undergraduate major and cumulative grade point average (GPA), domestic or international undergraduate degree, prior graduate degree and type, Graduate Record Exam (GRE) verbal, quantitative, and analytical writing scores, GRE physics subject test scores, TOEFL scores for international applicants, as well as whether the students were traditional graduate students (i.e., matriculated immediately following prior education without intervening work experience). Undergraduate degree majors were categorized as ‘physics', ‘engineering', or ‘other'. Degrees from the United States and Canada were categorized as ‘domestic’ and all others as ‘international'. Prior graduate degrees were categorized as ‘MS in physics', ‘PhD in physics', ‘MS in Engineering', ‘PhD in Engineering', ‘MS other', or ‘PhD other'. Students were also evaluated based on whether they were admitted into the PhD program or the MS program. Approximately 2‐3 and 12‐13 students are admitted annually into the PhD and MS programs, respectively; thus the admissions process is more competitive for PhD positions and should result in higher quality admissions statistics for those matriculating into the PhD program.

During our admissions process, the members of the admissions committee review all applicants that meet our program prerequisites. Each application file includes undergraduate major and cumulative GPA, prior graduate degree, type, and GPA, transcripts for all prior educational institutions, GRE verbal, quantitative, and analytical writing scores, GRE physics subject test scores, TOEFL scores, letters of recommendation, employment history, any relevant medical physics experience, a statement of career objectives, and any other relevant qualifications or honors. Applicants are then ranked by the admissions committee as a group in order of preference based on these criteria. This ranking reflects the aggregate preferences of the admissions committee, as each applicant will be reviewed and evaluated through discussion among the group. From this overall admissions ranking, the appropriate number of students are accepted into the program each year. An important question for us was whether this ranking was correlated with student success. In other words, are we basing our admissions decisions on metrics that are valid predictors of student success?

A number of established metrics have been used to gauge graduate student success, including GPA, first year graduate GPA, degree attainment, time to degree, scores on comprehensive exams, dissertation quality (for PhD students), professional accomplishments (e.g., publications, citations, etc.), rating scales, and performance work samples.^2^, ^3^ As described here, we employed some, but not all, of these metrics, as well as others which we found to be valuable indicators of success in our particular program. The success metrics used for this study were first year graduate GPA, degree attainment, final score in two difficult courses in the program (considered “weed‐out” courses), performance in oral review sessions, score on the program exit examination, and faculty rating. Performance in the program oral review sessions was subjectively rated on a scale of 1‐3 from high to low. These sessions test the student's knowledge of various topics in clinical medical physics, their ability to assimilate didactic knowledge from coursework in the program and apply it to practical clinical problems, and their ability to answer questions under pressure in oral form. As such, these sessions test the qualities that are representative of successful candidates for board certification taking the American Board of Radiology (ABR) oral examination. The program exit examination is a multiple choice exam modeled after the written components of the ABR certification examination. The exit exam covers all aspects of the program and is taken immediately prior to graduation by all MS students. PhD students do not take the exit examination; thus the statistics for this metric are only relevant for MS students. The faculty rating is a subjective aggregate rating of the student's expected career success. Faculty raters were asked to consider general intellectual capability, math and physics capabilities, written and verbal communication skills, independence/initiative, and work ethic/desire to achieve. In essence, the faculty rating was representative of the relative desire to hire this student for either a clinical medical physics position (for MS students) or an academic medical physics position (for PhD students). This rating was performed by five program faculty members using a 1‐5 scale from high to low, and average scores were calculated for each student. Not all faculty members had enough knowledge of each student to provide a rating. All rating was performed retrospectively at the end of the period of study. Finally, the faculty rating was reduced to a binary outcome with 1.0<X<2.5 for category 1 and 2.5<X<5.0 for category 2. A cutoff value of 2.5 was chosen since this was the median value for average faculty rating. With only 108 students, evaluating correlations for each of the faculty rating categories individually would likely not have produced statistically meaningful data. We chose not to include postgraduation (professional accomplishment) performance metrics such as publications, citations, and attainment of board certification since many of the graduates have not been in the workforce long enough to build a significant publication or citation base or to become eligible for the final section of their board certification exam.

We defined a “traditional” student as one who had no breaks of one calendar year or more between successive educational programs. Students who had breaks of one calendar year or more during which they obtained professional work experience were labeled as “nontraditional”. Origin of undergraduate degree was separated into either “USA/Canada” or “other”. All paper‐based TOEFL scores were converted to corresponding computer‐based scores using the Educational Testing Service score comparison tables.

MS and PhD applicants have been pooled together in this study to increase the statistical strength of the sample size. This requires justification since MS and PhD students may have different backgrounds, program expectations, and career goals. Characteristics of the admissions statistics and backgrounds (traditional vs. nontraditional, domestic vs. international, and previous graduate degrees) of students matriculating into the MS and PhD programs are very similar, as presented in the results section. In fact, many of our matriculating students applied to both programs. Faculty rating inherently takes into account differences in expectations and career goals, the exit exam is explicitly only for MS students, and course average and first year graduate GPA are objective metrics which do not differ between the two groups since both groups take identical coursework for the first year. Given these similarities in admissions statistics and outcome metrics, it seems reasonable to pool the data for both groups.

Data was analyzed using SAS version 9.2 (SAS Inc. Cary, NC, USA). The “PROC Means” procedure was used to generate basic demographic statistics, including the calculation of means, medians, standard deviations, and ranges for all variables. Categorical variables for GRE scores were created using median values as the cutoff. These categorical variables were separated as above/below median value. The dichotomized faculty rating performance variable was analyzed using paired *t*‐tests and logistic regression. All other performance variables were analyzed using paired *t*‐tests, ANOVA, and Linear Regression.

## III. RESULTS

Student demographics and admissions statistics are listed in Table 1. Quantitative and analytical GRE scores are given in the scale used by the Educational Testing Service (ETS) at that time, but can be converted to the new scale using concordances available through the ETS.

**Table 1:** Admissions statistics and academic background information for 108 students matriculating into the Wayne State University MS and PhD programs in Medical Physics

	*Admissions Statistics*											
	*Mean*			*Median*			*Max*			*Min*		
*Variable*	*MS*	*PhD*	*Combined*	*MS*	*PhD*	*Combined*	*MS*	*PhD*	*Combined*	*MS*	*PhD*	*Combined*
Undergraduate GPA	3.50	3.60	3.51	3.50	3.60	3.53	4.00	4.00	4.00	2.37	3.13	2.37
GRE Verbal	523	573	531	540	570	550	800	670	800	310	360	310
GRE Quantitative		774	753	760	780	760	800	800	800	590	710	590
GRE Analytical/Writing	4.3	4.3	4.3	4.5	4.5	4.5	6.0	6.0	6.0	2.5	2.5	2.5
GRE Subject (physics)	765	810	774	800	790	795	920	920	920	600	720	600
TOEFL	246	246	246	250	250	250	280	267	280	203	213	203
	*Physics*			*Engineering*			*Other*					
Undergraduate Degree	71			20			17					
	*MS Physics*			*PhD Physics*			*MS/PhD Engineering*			*MS/PhD Other*		
Previous Graduate Degree	17			15			15			9	

Scores presented here can also be converted to percentiles using these concordances. Binary categorical variables are not listed in Table 1, but are described here. Of the 108 students, 93 were accepted into the MS program and 15 into the PhD program. These matriculation rates maintain a total number of students in each program at approximately 10 and 24 for the PhD and MS programs, respectively, with these enrollment levels dictated by research funding levels and faculty/clinical resources for the PhD and MS programs, respectively. According to our definition, 77 (71%) matriculating students were identified as “traditional”, while the remaining 31 (29%) were identified as “nontraditional”. The fraction of traditional students was similar for both the PhD (80%) and MS (70%) programs. Sixty‐five matriculating students completed their undergraduate degree in the USA or Canada, while 43 completed it in another country. The fraction of matriculating students from the USA or Canada was similar for both the PhD (53%) and MS (61%) programs. Of the 108 matriculating students during this period, 86 graduated, 10 left the program, and 12 are still progressing toward a degree. All ten of the students who left the program were enrolled in the MS program. Fifty‐six students (52%) entered the MS or PhD program with at least one prior graduate degree (specific disciplines listed in Table 1) compared with 52 (48%) with no prior graduate degree. The fraction of students matriculating into the PhD program with at least one prior graduate degree was slightly higher (67%) than that of students matriculating into the MS program (47%). However, the fraction of PhD matriculants with a prior PhD in physics (13%) was essentially the same as that of MS matriculants (15%). This is expected to change with the introduction of graduate certificate programs.

Student performance metrics are listed in Table 2. First year Graduate GPA (GGPA) is used as the coursework metric rather than overall GPA since it is comprised of core didactic courses within our program, rather than electives and research coursework for which the GPA is generally higher. Thus, disparities in GPA are best observed through evaluation of the first year GPA. The variable “course average” is the average final score from the two courses mentioned in the Methods Section above. Table 3 lists statistical correlation data for admissions statistics against four performance metrics: faculty rating, course average, first‐year GGPA, and exit exam.

**Table 2:** Outcome statistics for 108 students matriculating into the Wayne State University MS and PhD programs in Medical Physics, including first year graduate GPA, average of two “weed‐out” courses, MS program exit exam, oral review session rating, and postgraduation rating by multiple faculty members

		*Outcome Statistics*		
*Variable*	*Mean*	*Median*	*Max*	*Min*
1^st^ year GGPA	3.67	3.70	4.00	2.29
Course average	86.0	87.5	98.8	57.6
Exit exam (MS only)	70.1	70.0	94.0	43.0
Oral review session rating	1.6	1	3	1
Faculty rating	2.5	2	5	1

**Table 3:** Statistical p‐values for evaluation of the potential correlation of admissions statistics listed below against four performance metrics: postgraduation faculty rating, average in “weed‐out” courses, first year graduate GPA, and MS exit exam

*Admissions Statistic*	*Faculty Rating*	*Course Average*	*1^st^ Year GGPA*	*Exit Exam*
Undergraduate degree ‐ physics vs. engineering	0.020^a^	0.579	0.161	0.656
Undergraduate degree ‐ physics vs other	0.787	0.251	0.609	0.687
Undergraduate GPA	0.998	0.430	0.941	0.592
GRE verbal	0.208	0.048^a^	0.030^a^	0.009^a^
GRE quantitative	0.345	0.0003^a^	0.467	0.003^a^
GRE analytical writing	0.064^b^	0.534	0.070^b^	0.750
GRE physics	0.008^a^	0.793	0.647	0.763
GRE verbal categorical (cutoff=550)	0.572	0.074^b^	0.068^b^	0.065^b^
GRE quantitative categorical (cutoff=760)	0.540	0.074^b^	0.293	0.002^a^
GRE analytical writing categorical (cutoff=4.5)	0.245	0.539	0.236	0.786
TOEFL	0.025^a^	0.023^a^	0.005^a^	0.120
Undergraduate country	0.006^a^	0.929	0.395	0.996

The p‐value is from chi‐squared tests for categorical variables and t‐tests for continuous variables.

a ^a^ P‐values less than 0.05 are considered statistically correlated.

b ^b^ P‐values between 0.05 and 0.10 indicating statistical trends.

### A. Faculty rating

“Faculty rating” represents the binary categorical value (i.e., whether the average faculty rating is greater than or less than 2.5). While a clear trend was observed between faculty rating and GRE analytical writing score (p=0.064), none of the general GRE sections were statistically correlated with faculty rating. The physics subject GRE score was correlated with faculty rating (p=0.008); however, there were only 14 such scores gathered within the group, since the physics subject GRE is not required for admission. While undergraduate GPA was not correlated with faculty rating (p=0.998), undergraduate degree type was. Students with undergraduate degrees in engineering had a statistically significantly higher faculty rating (p=0.020), while there was no statistically significant difference between those with a physics degree and those with an undergraduate degree classified as “other” (p=0.787). We also evaluated the GRE sections categorically with cutoff values given in Table 3 and none of these were correlated with faculty rating. The only other statistically significant correlation for faculty rating was with undergraduate country (p=0.006), with students from the US and Canada rated higher than those from other countries.

### B. Course average, first year GGPA, exit exam score

The GRE quantitative score, while not statistically correlated to faculty rating (p=0.345) or first‐year GGPA (p=0.467), was correlated with both course average (p=0.003) and exit exam score (p=0.003). More surprising was the correlation between GRE verbal score and course average (p=0.048), first‐year GGPA (p=0.030), and exit exam (p=0.009). In addition to the trend with faculty rating, the GRE analytical writing score showed a strong trend toward correlation with the first‐year GGPA (p=0.070). The only categorical GRE correlation was between GRE quantitative score and exit exam (p=0.002). The course average, first‐year GGPA, and exit exam score were not statistically correlated with an undergraduate degree in physics, engineering, or “other”. One other interesting correlation was between TOEFL score and first‐year GGPA (p=0.011). While faculty rating was strongly correlated with undergraduate country (p=0.006), none of the other three performance metrics were correlated with undergraduate country (p>0.395).

Table 4 lists additional relevant correlations between performance metrics and admissions statistics. These results show no correlation of faculty rating with traditional/nontraditional student status (p=0.234); however, there is a statistically significantly higher rating for students accepted into the PhD program (p=0.040). We found no statistical correlation between admissions rank and undergraduate country (p=0.451). Faculty rating was found to be strongly correlated with course average (p<0.0001), first‐year GGPA (p<0.0001), exit exam (p=0.002), and oral review score (p<0.0001). As expected, course average was strongly correlated with exit exam score (p=0.001). A trend toward domestic students scoring higher on the GRE verbal exam (p=0.099) was observed, while statistically significant differences were observed for the quantitative and analytical writing sections. Domestic students scored better on the analytical writing section (p=0.008), while nondomestic students scored better on the quantitative section (p=0.003). All performance metrics evaluated in Table 4 were strongly correlated with admissions rank, namely faculty rating, course average, exit exam score, and first‐year GGPA (p=0.002, <0.0001, 0.046, and 0.003, respectively).

**Table 4:** Additional statistical correlations investigated in this study along with p‐values from chi‐squared tests

*Statistical Comparison*	*P‐value*
Faculty rating vs. Program	0.040^a^
Faculty rating vs. Traditional	0.234
Faculty rating vs. Undergraduate GPA categorical (cutoff=3.0)	0.644
Faculty rating vs. Course average	<0.0001 ^a^
Faculty rating vs. 1^st^ year GGPA	<0.0001 ^a^
Faculty rating vs. Exit exam	0.002^a^
Faculty rating vs. Oral review	<0.0001 ^a^
Course average vs. Exit exam	0.001^a^
GRE verbal vs. Undergraduate country	0.099^b^
GRE quantitative vs. Undergraduate country	0.003^a^
GRE analytical writing vs. Undergraduate country	0.008^a^
Oral review vs. Undergraduate country	0.062^b^
Admissions rank vs. Faculty rating	0.002^a^
Admissions rank vs. Course average	<0.0001 ^a^
Admissions rank vs. Exit exam	0.046^a^
Admissions rank vs. 1^st^ year GGPA	0.003^a^
Admissions rank vs. Undergraduate country	0.451

a ^a^ P‐values less than 0.05 are considered statistically correlated.

b ^b^ P‐values between 0.05 and 0.10 indicating statistical trends.

## IV. DISCUSSION

It is interesting to note that undergraduate GPA and quantitative GRE score, two metrics which typically garner significant consideration within the admissions process, were not statistically correlated with faculty rating. Faculty rating is the performance metric we felt was most relevant since it is probably the strongest predictor of future professional success gathered within this study. One might have expected the quantitative GRE score to be strongly correlated with faculty ranting. However, the statistical spread in quantitative GRE scores was relatively narrow. We speculate that since almost all matriculating students had very high quantitative scores, it was difficult to draw any statistical inferences. A similar observation may be made with respect to undergraduate GPA. Stated succinctly, given a set of students with high undergraduate GPA and quantitative GRE scores selected through our admissions process, there appears to be no additional correlation with success within this narrow range of GPA and GRE scores.

In the interest of determining whether there is a quantitative GRE score cutoff below which there is a statistically significant correlation with success, we evaluated this score as a binary categorical variable above or below 700 and reevaluated it. While there was still no statistical correlation (p=0.334), this could be a consequence of the limited data. Only 10 out of 108 matriculants had a quantitative GRE score below 700. While many faculty members in medical physics graduate programs likely view the quantitative score as the most important score, the fact that the GRE analytical writing score appeared to be most correlated with faculty rating may not be surprising when one considers the functions that this section tests. The ability to interpret information, make analytical decisions about it, and communicate those decisions to others represents a very important quality in a medical physicist both as a scientist and as a clinician. All of our matriculants have high quantitative GRE scores, but the large disparity in analytical writing capabilities is evidently the most important standardized score differentiator for faculty rating for the cohort of students that we accept into our program.

An undergraduate degree in physics was not statistically correlated with increased success when compared to students with other degrees. In fact, engineering students score statistically higher in the faculty rating process, although this correlation was not observed in the other performance metrics. This may be due to the fact that many of the engineering students entering our program come from nuclear engineering or other engineering disciplines which provide excellent preparation for medical physics graduate education. Indeed, even those coming from undergraduate degrees in the “other” category had strong physics and math backgrounds. It should be noted that the physics prerequisites for our program throughout the study period were consistent with those currently required by the ABR for entry into the certification process. The fact that undergraduate GPA is not correlated with success is likely due not only to the relatively narrow spread of undergraduate GPAs for students we select through the admissions process, but also to the fact that the meaning of this metric varies so widely for different disciplines and institutions. Physics has traditionally been one of the disciplines least affected by grade inflation, and the amount of grade inflation almost certainly varies between institutions and undoubtedly varies between different disciplines. As a result, a GPA below the matriculating student median of 3.53 for a physics degree from a particular university may be an impressive achievement and could be more difficult to attain than a GPA much higher than the median in a different discipline or from a different university. One might expect that students with a very low undergraduate GPA would be less likely to succeed; however, we compared faculty rating with GPA above and below 3.0 and found no statistically significant correlation (p=0.558). This may be due to the fact that there were so few such students in our cohort (only 13 out of 108) and that such students had outstanding standardized test scores and other admissions statistics that helped them gain admission despite a poor GPA. Thus, these students were clearly very capable of success even though they did not achieve a high undergraduate GPA. This highlights the value of placing emphasis on an appropriate variety of factors in the admissions process rather than eliminating candidates from consideration based on a single poor statistic.

Perhaps the most interesting statistical correlation was between faculty rating and undergraduate country. This result was not necessarily surprising on its own, but was intriguing considering there was no statistical correlation between undergraduate country and any of the other performance metrics. As a reasonability check, we investigated the correlations between faculty rating and course average, first‐year GGPA, exit exam, and oral review score, and found them all strongly correlated (p<0.002). This is to be expected as faculty rating is (and should be) significantly influenced by these parameters. We then evaluated all GRE sections to determine whether there was a correlation with undergraduate country, and these correlations are observed in Table 4 and described in the Results section above.

The course average metric is based on examination scores in two “weed‐out” courses in the program, and thus is likely most strongly correlated with quantitative capabilities. The faculty rating was shown to be uncorrelated to quantitative capabilities as measured by the GRE exam for this group of students. On the other hand, faculty rating considers many other qualities, specifically including those tested by the GRE analytical writing section, such as the ability to assimilate and communicate information. We conclude that since nondomestic students have higher quantitative GRE scores, they are able to score as well as domestic students in the “weed‐out” courses, even with a potential language barrier. However, domestic students have an advantage in analytical writing capabilities over nondomestic students and thus fare better in aspects of training that require analytical and communication skills. If we were able to statistically evaluate faculty rating separately for only those matriculants who achieved high scores on the GRE analytical writing test, we expect that there would be no correlation with undergraduate country. Unfortunately, there were only six nondomestic students who scored higher on the GRE A/W test than the overall median score of 4.5, and any statistical inferences from this small data set would be relatively weak as a result. Anecdotally, four of the six nondomestic students with GRE A/W scores greater than 4.5 were rated at or above the mean faculty rating. While the oral review session score was not statistically correlated with undergraduate country, there was a very strong trend (p=0.062). Of course, while statistics such as those presented here can provide meaningful group trends, caution must be exercised in applying such information to individual applicants.

Taken as a whole, the results here indicate that predicted success in medical physics is based on more than quantitative capabilities, and that other skills such as oral and written communication play a major role in predictions of future success by our medical physics program faculty members. The fact that a language barrier could potentially affect the future success of our graduates is a significant source of concern for our program. In situations in which it is clear that a language barrier is hindering a student's performance, the student is referred to the English Language Institute (ELI) at WSU. The ELI provides intensive language programs in English communication, cultural orientation, and academic preparation for nonnative English speakers. Another mechanism helping to remediate this issue is the provision of videotaped course lectures. Several of our courses now offer online streaming audio and/or video of each lecture, including the two courses considered in the “course average” metric used here. Students with language difficulties may have trouble keeping pace with the live lecture, but may now watch it as many times as necessary to comprehend the material. We hope that these resources will help alleviate the influence of language difficulties and educational culture on the success of our international students.

The purpose of the admissions process is to determine which applicants are best suited for success in our program and in the profession. Students who succeed in the “weed‐out” courses will succeed in our program. Of the ten students in this cohort who did not complete a degree, six failed one or both of these courses and four passed both but decided to leave the program of their own accord to pursue other opportunities. No student who passed both of these courses failed out of the program for any other reason. Thus course average is clearly an indicator of student success as measured by progression to degree. The fact that course average, exit exam score, and first‐year GGPA were strongly correlated to admissions rank (p<0.0001, 0.046, and 0.003, respectively) is evidence that the ranking performed during our admissions process is a good predictor of student success in our program coursework. In reviewing the students who did not progress to degree, there were no clear trends exhibited which would allow us to better select students who will complete their degree. However, with an attrition rate of only approximately 9%, it is difficult to determine such trends.

Although there was a correlation between faculty rating and undergraduate country (p=.006), there was no correlation between admissions rank and undergraduate country (p=0.451); thus, there currently appears to be no bias in selecting domestic vs. international students within the admission process. Finally — and maybe most importantly — faculty rating was found to be strongly statistically correlated to admissions rank (p=0.002). So we can conclude that, while only two of the admissions statistics we evaluate during the admissions process appear to be independently correlated with predicted success (undergraduate degree type and physics subject GRE score), the metrics we feel are the most relevant predictors of success in our program and in a professional career, first‐year GGPA and faculty rating, respectively, are strongly correlated with our admissions ranking (p=0.003 and 0.002, respectively). Another example of this correlation is observed in the significantly higher faculty rating of PhD matriculants vs. MS matriculants (p=0.040). Many students matriculating into the MS program applied to the PhD program also but were not ranked as high during the admissions process as the PhD matriculants for that year. Table 1 also illustrates the stronger admissions statistics observed for the PhD matriculants in comparison to the MS matriculants. The fact that the PhD matriculants have a statistically higher faculty rating reaffirms the correlation between our admissions process and predicted success. Thus the question of whether we are basing our admissions decisions on metrics that are valid predictors of future success has been answered affirmatively.

Three trends were observed in the demographics of matriculating students over this time period. The fraction of matriculants with undergraduate degrees in physics and the fraction completing their undergraduate degree in the US or Canada both increased, while the fraction with prior graduate degrees decreased. From 2004‐2007, 30 of 55 (55%) of matriculating students had an undergraduate degree in physics, and 28 of 55 (51%) completed their undergraduate degree in the US or Canada. In contrast, from 2008‐2011, 41 of 53 (77%) of matriculating students had an undergraduate degree in physics, and 37 of 53 (70%) completed their undergraduate degree in the US or Canada. From 2004‐2007, 34 of 55 (62%) of students matriculated with at least one prior graduate degree, compared to 22 of 53 (42%) of students matriculating from 2008‐11. We speculate that these trends may be due to more widespread knowledge of the existence of the profession among undergraduate students in physics, particularly among those in the US and Canada. One might expect more widespread knowledge of the profession to also result in a decrease in the number of nontraditional students entering the program, however this trend was not observed. Fifteen of 55 (27%) matriculants from 2004‐07 were classified as “nontraditional” compared to 16 of 53 (30%) matriculants from 2008‐11. Trends toward decreasing GRE A/W score and undergraduate GPA were observed over this time period, with slopes of −0.15 points per year and −0.04 points per year, respectively. However, there was no statistically significant decrease in undergraduate GPA for matriculating students with a degree in physics. The decrease in GRE A/W score for domestic students (slope=−0.09) was far smaller than the decrease for international students (slope=−0.37). There was also a decreasing trend in TOEFL scores, with a slope of −3.4 points per year. The downward trends in TOEFL and international student GRE A/W scores appear to indicate that the English‐speaking skills of our matriculating international students are decreasing. No other statistically significant trends in either admissions or outcome metrics were observed over this time period. Changes in our admissions statistics are likely due to changes in the applicant pool resulting from significant changes within the medical physics educational infrastructure over this time period. The number of CAMPEP‐accredited graduate programs in medical physics increased from 10 to 37 over the time period of this study. While the number of applicants to our graduate program has not decreased significantly over this time period, the rapid proliferation of accredited programs has certainly had an effect on the applicant pool for all programs.

This study has resulted in one change to our admissions process thus far. The GRE Physics subject test was previously strongly recommended only for PhD applicants, but is now strongly recommended for all applicants. We have no plans to offer additional preference to domestic applicants or those with engineering degrees. While faculty rating was correlated with both of these parameters, it is a subjective metric which could be susceptible to bias, and there were no correlations identified for the objective metrics (such as first year graduate GPA) or course average. Since the admissions ranking is so strongly correlated to success metrics studied here, we do not feel compelled to make significant changes at this time.

## IV. CONCLUSIONS

The results here suggest that an undergraduate degree in physics should not be considered a fundamental requirement for entry into our program. They also suggest that success in medical physics is based on more than quantitative capabilities, since analytical and communication skills appear to play a role in both success in our program and future success predicted by our faculty members. This is highlighted by the fact that the GRE analytical writing score is the standardized test score most strongly correlated to success in our medical physics graduate program, as determined by faculty rating at the end of the program. It is also indicated by the fact that English language capabilities are correlated to faculty rating, course average, and first year graduate GPA through the TOEFL score. Other admissions statistics that were anticipated to be correlated with student success, such as undergraduate GPA and quantitative GRE score, were not found to be correlated with faculty rating (p=0.998 and 0.345, respectively). However, the quantitative GRE score was correlated with student performance in the “weed‐out” courses and with the exit exam. It is important to note that a high degree of selection on undergraduate GPA and quantitative GRE score takes place through the admissions process. This results in a relatively small statistical spread in these two metrics, and the small remaining variations are evidently not statistically meaningful. As such, these results do not necessarily imply that one should not strongly consider traditional metrics, such as undergraduate GPA and quantitative GRE score, during the admissions process. They suggest that once applicants have been initially filtered by these metrics, additional selection should be performed via the other metrics shown here to be correlated with success. In addition, the relatively small sample size makes it difficult to draw concrete conclusions about how to apply this information to the admissions process.

While only two parameters used for evaluation in our admissions process (physics GRE and undergraduate degree) are independently correlated with faculty rating, our overall admissions rank is strongly correlated (p=0.002). When taken as a whole, the parameters used to make admissions decisions for our program are very accurate in predicting both success within our program and expected success after graduation. This is illustrated by the very strong statistical correlation between admissions rank and course average (p<0.0001), first year graduate GPA (p=0.003), and faculty rating (p=0.002). This study has confirmed that our current admissions process is very effective in identifying candidates who will be successful in our program and are expected to be successful after graduation. It also provides insight into the relative importance of the factors upon which we base our admissions decisions, and this insight will help guide future admissions decisions.
